# Exploring prostate cancer screening among men in Accra using the health belief model

**DOI:** 10.4314/gmj.v57i3.10

**Published:** 2023-09

**Authors:** Isaac M Boafo, Peace M Tetteh, Rosemond A Hiadzi

**Affiliations:** University of Ghana, School of Social Sciences, Department of Sociology, Legon, Ghana

**Keywords:** Prostate cancer, cancer screening, Health Belief Model, men's health

## Abstract

**Objective:**

To explore the prevalence of prostate cancer screening among Ghanaian men and interrogate why some individuals screen for the disease and others do not.

**Design:**

A cross-sectional questionnaire survey based on the Health Belief Model was used to collect data from 356 men aged 40 years and above. Data were collected between February and March 2021.

**Setting:**

The study was conducted in the Accra metropolitan area of the Greater Accra region of Ghana.

**Participants:**

Convenience sampling was used to recruit participants for the study.

**Results:**

Although 86% of the respondents had heard about prostate cancer, only 23% had ever screened for it. Logistic regression analysis suggested that knowledge of the disease (OR = 1.19, CI 95% = 1.03 -1.38) and barriers to screening (OR = .87, CI 95% = .83 -.91) were statistically significant predictors of screening behaviour.

**Conclusion:**

HBM has limited predictive power as far as our study is concerned. We suggest increasing public education on prostate cancer and its screening methods. The cost of screening should also be made more affordable so as not to become a barrier.

**Funding:**

None declared

## Introduction

Prostate cancer is becoming a public health issue globally.^1^ Following the disease transition in many developing countries 2, cancer has become one of the leading causes of death in African countries, including Ghana.^3^,^4^ Generally, the incidence rate of prostate cancer is lower in developing countries than in developed countries; however, mortality rates are higher in developing countries than in developed countries.^5^ For instance, in the UK, the incidence rate of prostate cancer in 2012 was 111.1, and the mortality rate was 22.8 per 100,000. In contrast, Uganda had an incidence rate of 48.2 and a mortality rate of 38.8 per 100,000. In Burkina Faso, the incidence rate was 19.1, and the mortality rate was 18.8 per 100,000 6. The GLOBOCAN report even suggests that the higher prevalence of prostate cancer in more developed regions may be due to the widespread uptake of prostate specific antigen testing (PSA) and subsequent biopsies.^7^

Indeed, the lower incidence rates in developing countries may not truly reflect the situation as the non-testing and non-disclosure of such diseases are prevalent.^8^ Thus, the rates might be worse than is known.

These statistics demonstrate the need for adequate screening programs in developing countries, particularly in Africa, as sufficient evidence suggests that detecting the disease early helps its successful treatment and reduces the associated mortality rates. Moreover, available research suggests that men of African descent are at a higher risk of prostate cancer 9, thus making screening imperative for African men, particularly those above 45.^10^ Though experts disagree on the prudence of prostate cancer screening, most experts agree that at-risk men must be screened.^11^,^12^ For instance, the American Cancer Society recommends that prostate cancer screening consisting of a digital rectal examination and prostate-specific antigen be offered annually to men aged 50 years and above who have 10 years or more of life expectancy but an earlier age of 45 years recommended for African American men.^13^,^14^

Despite this evidence, prostate cancer screening is low among West African men 8,15,16. Consequently, studies in West Africa report that prostate cancer is diagnosed at advanced stages.^17^,^18^ Several factors have been found to account for the low practice of prostate cancer screening among West African men.

In Burkina Faso, Kabore, Kambou, Zango, Ouédraogo 19 found that out % of 600 respondents, 62% did not know about the prostate gland or prostate cancer. Additionally, while 70% of the men were unaware of any diagnostic tests for prostate cancer, 12% of men cited too much sexual activity as a major risk factor for prostate cancer. Several other studies in Africa 20-22 have also reported low knowledge as a reason for the low screening rates. Other factors responsible for non-testing include embarrassment, cost, concern about abnormal test results or cancer 23, perceived vulnerability and taboos 24. However, these barriers do not always hang out together logically, for instance, one may reasonably expect people with high knowledge of the disease to undertake screening, however, some studies have found low rates of screening despite participants' high knowledge of prostate cancer and the benefits of screening.^25^

Despite the high morbidity of prostate cancer in Ghana, with studies reporting 32 per 100,000 population or more^26^, about three-quarters of prostate cancer cases are reported at health facilities in advanced stages.^27^,^28^ Low knowledge, poor perceptions about prostate cancer, and the availability of alternative therapies are reasons for late reporting of prostate cancer screening and treatment.^29^-^31^ A survey conducted at the Korle Bu Teaching Hospital in 2007 suggested that 800 out of 1000 men diagnosed with prostate cancer died, and the country recorded 200 cases out of every 100,000 men.^32^ According to Raphael Obu 32, many male patients aged 40 years and above complain of weak urinary stream, frequent and urgent urination, a feeling of not being able to empty their bladder completely and a total loss of libido. The author argues that while these observations are increasing, little effort has been made to increase awareness for early detection and treatment. Arguably, increased prostate cancer screening is desperately needed. However, it remains a fact that there is data paucity on prostate cancer in Ghana and the factors which influence screening practices.^26^,^31^ This study, therefore, utilizes the Health Belief Model to ascertain the predictive factors for prostate cancer screening among Ghanaian men.

HBM is a cognitive model used in identifying and predicting health behaviours.^33^ The model originally formulated by Rosenstock has four main constructs: perceived susceptibility, perceived severity, perceived benefits and perceived barriers. HBM postulates that if people believe that they are at risk of a health condition, and they perceive that condition to be serious or very harmful, then they are likely to engage in the behaviour that helps to avoid that condition provided the benefits of that behaviour outweighs the barriers or cost of that action. Barriers could be financial, social and cultural.^34^

In this study, prostate cancer screening was the health behaviour of interest. Per the model, we expected that if people believed they were at risk of prostate cancer. They deemed the disease as a serious one. Then they would screen for it provided they believed they were not faced with barriers they consider difficult to navigate and believe that screening would benefit early detection and treatment of prostate cancer and thus increase their chances of survival.

The Health Belief Model (HBM) has been intensively used for assessing health beliefs associated with cancer screening behaviours.^35^,^36^ Consequently, we used HBM as a framework since it places more emphasis on the prevention of prostate cancer. Perceived susceptibility, severity, benefits, and barriers were all investigated in the current study alongside knowledge of prostate cancer and screening. The following research questions drove the study:

- What are prostate cancer awareness and knowledge levels among men in Accra?
- How prevalent is prostate cancer screening among men in Accra?
- What are the predictors of prostate cancer screening/non-screening among men in Accra?

## Methods

### Study Site and Sampling

A cross-sectional questionnaire survey was conducted between February and March 2021 in the Greater Accra Region of Ghana. The Accra Metropolitan Area was randomly selected from the Region's 10 Municipal and Metropolitan areas.^37^ The cosmopolitan nature of the study site presented an opportunity for people from diverse socio-demographic backgrounds to be included in the study. According to the 2010 Population and Housing Census,^37^ of the Accra Metropolis had a male population of 887,673. Since this census was conducted ten years ago, the sample size was calculated on the entire male population instead of those aged 40 and above to cater for any increase in population. At a 95% confidence level and p-value of 0.05, the sample size was determined to be 384.^38^

Participants were conveniently recruited from shopping malls and stores, open markets, lorry parks and formal organisations within the metropolis, such as the ministries, banks, and insurance firms. The rationale behind selecting these sites was to ensure that the diversity of Ghanaian men regarding socio-demographic backgrounds was duly represented in the study as the study targeted the general public. To participate in the study, one had to be 40 years and above, must be a male and a Ghanaian.

In the markets and shopping malls, buyers and sellers who fit the eligibility criteria were invited to participate. In the lorry stations, passengers, transport owners, drivers, sellers and passers-by were all invited to participate in the study. In some formal organisations, some senior staff members offered to take some questionnaires and distribute them to other interested staff. This also resulted in some unqualified staff completing the questionnaire, as instructions might not have been spelt out. The data were collected using printed (hardcopy) questionnaires.

### Data collection instrument

The study utilized the HBM questionnaire for prostate cancer screening developed by Capik, Gözüm 39. The questionnaire comprises 41 items measured on a 5-point Likert scale anchored at 1=completely disagree and 5=completely agree. The questionnaire has four basic subscales, namely, perceived susceptibility (5 items), perceived severity (5 items), perceived barriers (15 items), and perceived benefits (7 items). To access the knowledge level of participants in relation to prostate cancer, Knowledge of Prostate Cancer Screening Questionnaire 40 was also utilized in the current study. The original questionnaire comprised 13 items with answer categories “true”, “false”, and “I don't know”.

Previous studies 36,41,42 have reported good reliabilities for the HBM and Knowledge of Prostate Cancer Screening questionnaires. These questionnaires have been used extensively, and their use in the current study is partly to ensure that the findings of this study can be compared with other studies globally.

### Data Organisation and Analyses

A simple sum score was computed for each of the constructs of the HBM. Perceived susceptibility to prostate cancer was made up of five items with a possible range of scores (PRS) of 5-25, perceived severity of prostate cancer consisted of five items (PRS = 5 to 25), perceived benefits of screening consisted of seven items (PRS = 7 to 35), and perceived barriers to screening consisted of fifteen items (PRS = 15 to 75).

Regarding the Prostate Cancer Knowledge Survey, responses were coded as ‘correct’ or ‘incorrect’. “I don't know” responses were also coded as ‘incorrect’. Each correct answer was assigned a score of 1, while an incorrect answer or “don't know” was awarded a score of 0. A simple sum score was computed to form the knowledge of the prostate cancer scale. The possible range of scores was 0-13, with a high score indicating high knowledge of prostate cancer.

Data were organised and analysed using Statistical Package for Social Sciences (IBM SPSS) version 22. Descriptive statistics were used in summarising and describing the data. Independent samples t-tests were used to examine the differences between those who have ever screened and those who have never screened for prostate cancer on selected continuous variables. Direct logistic regression was used to construct a predictive model for prostate cancer screening behaviour. All analyses were performed at a significance level of p≤ 0.05.

### Ethics approval and consent to participate

Generally, the study was guided by the ethical principles suggested in the Helsinki Declaration for conducting research with human subjects. Informed consent was taken verbally from the participants. The study was approved by the Ethical Review Board of the Noguchi Memorial Institute for Medical Research, University of Ghana (approval number 036/20-21).

## Results

### Socio-demographic characteristics of participants

A total of 400 questionnaires were administered. Of the 400 participants, 39 were below age 40, and 5 questionnaires were deemed invalid due to the number of missing cases. The analysis thus presented in this paper involves 356 participants. The ages of the 356 participants in the current study ranged from 40 to 80 years. The majority (53.7%) of the participants fell within the age range of 40-49. Analysis of the absolute ages showed that the mean age was 50.5 (SD = 8.65). The majority (79.2%) were married, and 71.1% reported employment.

Regarding educational attainment, about a quarter (25.2%) of the respondents had a first degree or higher. The socio-demographic characteristics of the respondents are presented in Table 1.

**Table 1:** Socio-demographic characteristics of respondents

Item Description	n (%)
**Age:**	
40-49	191 (53.7)
50-59	111 (31.3)
60-69	38 (10.7)
70+	16 (4.5)
**Religious Affiliation:**	
Christian	276 (77.5)
Muslim	69 (19.4)
Traditionalist	3 (.8)
Other religion	7 (2.0)
Marital Status:	
Single	43 (12.1)
Married	282 (79.2)
Divorced	14 (3.9)
Widowed	14 (3.9)
**Educational Attainment:**	
Less than primary	36 (10.1)
Primary	21 (5.9)
JSS/Middle School	78 (21.9)
Secondary School	79 (22.2)
Diploma	42 (11.8)
Bachelor's Degree	50 (14.0)
Postgraduate Degree	40 (11.2)
**Employment Status:**	
Employed	253 (71.1)
Unemployed	71 (19.9)
Retired	19 (5.3)

Source: Field survey 2021

Note: Percentages may not add to 100% due to missing values

### Prostate Cancer Awareness, Screening Behaviour, and Willingness to Screen

Although most (86%) of the respondents reported having heard about prostate cancer, only 23% had ever screened for prostate cancer. However, 79.2% of the respondents reported being willing to screen for prostate cancer if a healthcare provider or doctor recommended it. However, on their own, less than a third (32%) had clear intentions of discussing prostate cancer with their doctor or healthcare provider within the next 12 months. Data on prostate cancer awareness, screening behaviour and willingness to screen are presented in Table 2.

**Table 2:** Prostate cancer awareness, screening behaviour and intention to screen

Item Description	n (%)
Have you ever heard of prostate cancer?	
No	49 (13.8)
Yes	306 (86.0)
Have you ever screened for prostate cancer?	
No	272 (76.4)
Yes	82 (23.0)
Has anyone in your family been diagnosed with prostate cancer?	
No	202 (56.7)
Yes	42 (11.8)
I don't know	109 (30.6)
Do you have any intention of speaking to your healthcare provider about PCS in the next 12 months?	
No	235 (66.0)
Yes	114 (32.0)
Would you screen for prostate cancer if it is recommended by your doctor?	
No	32 (9.0)
Yes	282 (79.2)
May be	39 (11.0)
Would you be willing to let your healthcare provider perform DRE on you?	
No	69 (19.4)
Yes	233 (65.4)
May be	51 (14.3)
Would you be willing to undergo DRE if the healthcare provider is a woman?	
No	85 (23.9)
Yes	202 (56.7)
May be	64 (18.0)
Would you be willing to let your healthcare provider perform PSA on you?	
No	26 (7.3)
Yes	306 (86.0)
May be	21 (5.9)

Source: Field survey 2021

Note: Percentages may not add to 100% due to missing cases

Bivariate analyses suggested that people who did not know any family member with prostate cancer were less likely to have screened for X^2^(N = 352, df = 2) = 6.694, p = 0.035. Similarly, such respondents were also less likely to have intentions of discussing prostate cancer with their doctor or healthcare provider X^2^(N = 347, df = 2) = 17.365, p = 0.000.

### Knowledge of Prostate Cancer

The results suggest that respondents had inadequate knowledge about prostate cancer. The mean of the scale was 4.492 (SD = 2.543), below the scale's mid-point. As shown in Table 3, although 69.4% of participants knew that prostate cancer might grow slowly in men, less than a third (31.5%) knew that a man could have cancer and not exhibit any symptoms. Furthermore, over half (55.6%) knew that a family history of prostate cancer suggests that one is likely to have prostate cancer himself. Again, most respondents (75.3%) did not know that frequent lower back pain could be a symptom of prostate cancer, and 77.5% erroneously thought that a doctor could tell which men may die from prostate cancer and which men will not be harmed by prostate cancer.

**Table 3:** Prostate cancer knowledge

Item Description	Incorrect n (%)	Correct n (%)
Men who have several family members with prostate cancer are more likely to get prostate cancer	198 (55.6)	154 (43.3)
A man can have prostate cancer and have no problems or symptoms	242 (68.0)	113 (31.5)
Younger men are more likely to get prostate cancer than older men	252 (70.8)	101 (28.4)
Frequent pain often in the lower back could be a sign of prostate cancer	268 (75.3)	85 (23.9)
Most 80-year-old men do not need prostate cancer screening	247 (69.4)	105 (29.5)
Some treatments for prostate cancer can make it harder for men to control their urine	192 (53.9)	160 (44.9)
Some treatments for prostate cancer can cause problems with a man's ability to have sex	173 (48.6)	181 (50.8)
Some treatments for prostate cancer can stop a man from ever driving a car	276 (77.5)	76 (21.3)
A doctor can tell which men may die from prostate cancer and which men will not be harmed by prostate cancer	276 (77.5)	76 (21.3)
An abnormal PSA blood test means I have cancer for sure	269 (75.6)	85 (23.9)
I can have cancer and have a normal PSA test	259 (72.8)	95 (26.7)
Prostate cancer may grow slowly in men	107 (30.1)	247 (69.4)
Black African men are more likely to get prostate cancer	251 (70.5)	103 (28.9)

Source: Field survey 2021

Note: Percentages may not add to 100% due to missing cases

### Health beliefs on prostate cancer

The four basic constructs of the Health Belief Model, namely, perceived susceptibility, perceived severity, perceived barriers and perceived benefits, were examined in the current study. A simple sum score was computed for the items measuring these constructs. The possible range of scores for the perceived susceptibility scale was 6 to 30, with a mean of 13.43 (SD = 5.24), suggesting that men in the sample generally did not consider themselves prone or susceptible to prostate cancer. Independent samples t-test indicated no statistically significant difference between those who have ever screened and those who have never screened for prostate cancer. One major finding of the bivariate analyses was that there was a statistically significant difference between men who have ever screened for prostate cancer and those who have never done that in relation to perceived barriers. Those who have screened present a lower mean (33.40, SD = 9.55) than those who have never screened (44.34, SD = 8.84), p = 0.001. Details of the constructs of the HBM are presented in Table 4.

**Table 4:** Comparison of Health Belief Model factors among those who have ever and those who have never screened for prostate cancer

Sub-scales	Score range	Total Mean (SD)	Ever Screened Mean (SD)	Never Screened Mean (SD)	t	P value
**Perceived Susceptibility**	6 – 30	13.43 (5.24)	13.63(5.20)	13.37(5.26)	-0.385	0.701
**Perceived Severity**	3 -15	10.48 (2.97)	9.91 (3.17)	10.66(2.89)	1.979	0.049*
**Perceived Barriers**	15 – 75	41.83(10.11)	33.40(9.55)	44.34(8.84)	8.922	0.000*
**Perceived Benefits**	7 – 35	28.18(4.93)	27.97(5.49)	28.25(4.76)	0.415	0.678

Source: Field survey 2021

* Significant at 0.05

### Predicting Prostate Cancer Screening Behaviour

Direct binary logistic regression was performed to predict the likelihood of undertaking prostate cancer screening (see Table 5). The overall model was statistically significant *X*^*2*^(df = 7, N = 356) = 83.81, p = 0.000. The model explained between 26.4% (Cox & Snell R square) and 40.3% of the variance in screening behaviour and correctly classified 82.1% of cases. The strongest predictor of prostate cancer screening was perceived barriers (OR = .87, 95% CI: .83 – .91, p = 0.000). The model further suggested that knowledge of prostate cancer was positively associated with screening behaviour. Men who had higher knowledge of prostate cancer had a higher likelihood of screening for the disease (OR = 1.19, CI: 1.03 – 1.39, p < 0.05). This model thus suggests that apart from the barriers that people face (perceive) with screening, none of the other factors in the HBM are related to screening behaviour as far as the sample is concerned.

**Table 5:** Predicting prostate screening behaviour – Direct logistic regression

Variable	Coefficient	S.E	Wald	df	p- value	Odds Ratio	95% CI.
*Age*	0.03	0.02	2.18	1	0.140	.1.03	0.99 -.1.01
*Education:*							
**Less than first degree**	reference	-	-	-	-	-	-
**First degree or higher**	0.43	0.39	1.21	1	0.272	1.54	0.72 – 3.29
*Knowledge*	0.18	0.07	5.49	1	0.019	1.19	1.03 – 1.38
*Perceived Susceptibility*	0.06	0.04	2.53	1	0.111	1.06	0.99 – 1.13
*Perceived Severity*	-0.11	0.07	2.40	1	0.122	0.90	0.79 – 1.03
*Perceived Barriers*	-0.14	0.02	40.22	1	0.000	0.87	0.83 - 0.91
*Perceived Benefits*	0.04	0.04	0.98	1	0.322	1.04	0.97 – 1.11
*Constant*	0.91	1.73	0.28	1	0.598	2.49	

Source: Field Survey 2021

## Discussion

The current paper established the determinants of prostate cancer screening among men in Accra, Ghana. The results suggested that knowledge of the disease is one of the main predictors of screening behaviour. Those with higher knowledge of prostate cancer and screening procedures were likelier to have screened for the disease. This finding is consistent with a similar study conducted in the Sunyani municipality in Ghana, where a positive correlation was found between knowledge and attitudes towards screening 31. It is also consistent with many other studies on the disease within and outside Africa 43-45. Overall, the knowledge level reported in the current study was deemed inadequate as the mean fell below the midpoint of the knowledge scale. This finding is somewhat consistent with another study^19^ conducted in Burkina Faso, which reported that 62% of the 600 participants did not know about prostate cancer. Indeed, many other African studies have reported low knowledge as the reason for the low screening rates and the concomitant high prostate cancer mortality rate on the continent 46-48.

This inadequate knowledge level may explain why only 23% of the participants in the current study had screened for the disease, although 86% reported having heard about it. It is, however, worth noting that it is also possible for men to have high knowledge of prostate cancer and screening procedures and still not screen for the disease 25. In such instances, other factors, such as it being recommended by a medical doctor, may help explain screening behaviour.^23^

The current study also investigated the ability of the four basic constructs of the health belief model (perceived susceptibility, perceived severity, perceived benefits and perceived barriers) to predict prostate cancer screening practices among our sample. The binary logistic regression model suggested that only the ‘perceived barriers’ construct of the HBM is statistically significantly related to screening behaviour. The predictive power of the HBM seems to be limited, at least as far as prostate cancer screening practices are concerned, and there seem to be inconsistencies in studies in relation to which constructs in the model can predict screening behaviour.^49^,^50^ For instance, among Iranian men aged 50 years and over, it was found that perceived seriousness (β = 0.35, p < 0.01) and perceived benefits (β = 0.19, p < 0.01) were the statistically significant predictors of prostate cancer screening behaviour^50^. In another study among 200 men in Haiti, only perceived benefits were found to be a statistically significant predictor of prostate cancer screening.^49^ Yet another study^51^ found all four basic constructs of the HBM to be statistically significantly related to prostate cancer prevention behaviours.

A qualitative study also among Black Americans aged 40 years and above, it was reported that factors such as prostate cancer knowledge, perceived susceptibility, and negative outcome beliefs were associated with screening behaviour.^52^

### Limitations

By the nature of our research design, causal inferences cannot be made from our findings. The sampling technique used in the study presented in this paper was essentially one of convenience. Therefore, the sample may not be representative of the population. Notwithstanding the convenience sampling, the findings provide useful lessons on prostate cancer screening prevalence. Finally, per Krejcie, Morgan 38 formula, the sample size was supposed to be 384. However, only 356 questionnaires were found to be valid for inclusion in the analyses. We are, however, of the view that this shortage should not influence the results in any significant way.

## Conclusion

The Health Belief Model has limited predictive power as far as our participants are concerned. However, the Health Promotion Unit of the Ghana Health Service and other non-governmental organisations must intensify public education on prostate cancer and its screening.
